# Author Correction: Molecular anchoring of free solvents for high-voltage and high-safety lithium metal batteries

**DOI:** 10.1038/s41467-025-56862-2

**Published:** 2025-02-12

**Authors:** Zhuangzhuang Cui, Zhuangzhuang Jia, Digen Ruan, Qingshun Nian, Jiajia Fan, Shunqiang Chen, Zixu He, Dazhuang Wang, Jinyu Jiang, Jun Ma, Xing Ou, Shuhong Jiao, Qingsong Wang, Xiaodi Ren

**Affiliations:** 1 Hefei National Research Center for Physical Sciences at the Microscale, CAS Key Laboratory of Materials for Energy Conversion, Department of Materials Science and Engineering, University of Science and Technology of China, Hefei, Anhui 230026 China; 2 State Key Laboratory of Fire Science, University of Science and Technology of China, Hefei, Anhui 230026 China; 3 Engineering Research Center of the Ministry of Education for Advanced Battery Materials, School of Metallurgy and Environment, Central South University, No.932 South Lushan Road, Changsha, Hunan 410083 PR China

Correction to: *Nature Communications* 10.1038/s41467-024-46186-y, published online 6 March 2024

In the version of the article initially published, the *y* axes in Fig. 6 were not normalized to the correct mass of the active material. As shown in Fig. 1, the *y* axes have now been normalized by 2 mg and 4 mg for panels a and b, respectively, and the figure legend has also been updated. These corrections have been made to the HTML and PDF versions of the article.

**Fig. 1:**
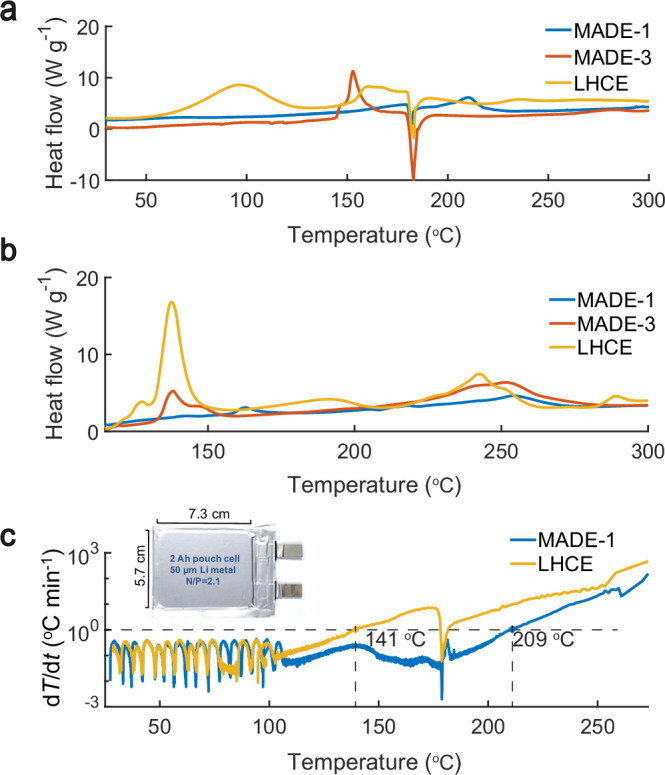
Original Fig. 6.

**Fig. 1:**
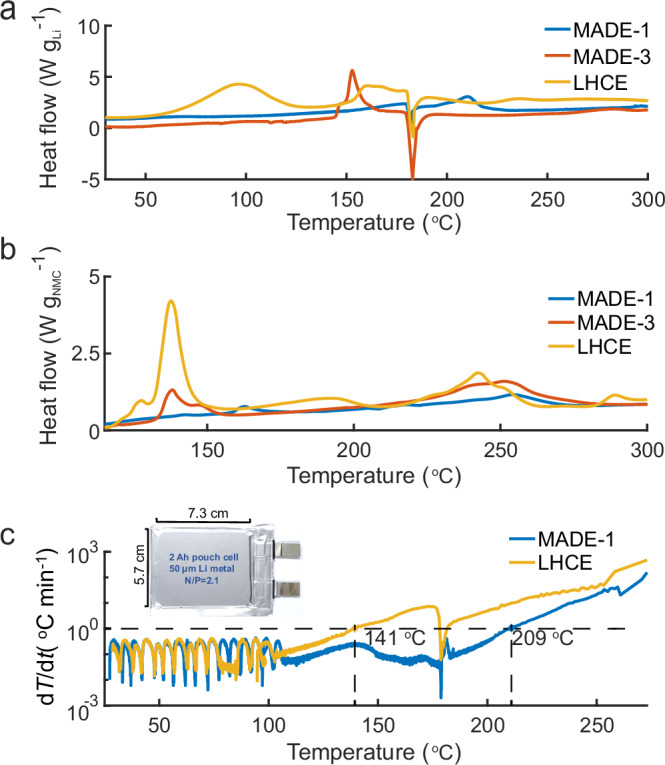
Corrected Fig. 6.

