# Supplementary material for: Molecular anchoring of free solvents for high-voltage and high-safety lithium metal batteries
Source: Nat Commun. 2024 Mar 6;15:2033. doi: 10.1038/s41467-024-46186-y (PMC10918083; doi:10.1038/s41467-024-46186-y)
Supplement: Supplementary file 1 — Supplementary Information [file 41467_2024_46186_MOESM1_ESM.pdf]

Supporting Information

Molecular Anchoring of Free Solvents for High-Voltage and High-Safety Lithium Metal Batteries

Zhuangzhuang Cui[1], Zhuangzhuang Jia[2], Digen Ruan[1], Qingshun Nian[1], Jiajia Fan[1], Shunqiang Chen[1], Zixu He[1], Dazhuang Wang[1], Jinyu Jiang[1], Jun Ma[1], Xing Ou[3], Shuhong Jiao[1], Qingsong Wang[2,*], Xiaodi Ren[1,*]

[1]Hefei National Research Center for Physical Sciences at the Microscale, CAS Key Laboratory of Materials for Energy Conversion, Department of Materials Science and Engineering, University of Science and Technology of China, Hefei, Anhui, 230026, China

[2]State Key Laboratory of Fire Science, University of Science and Technology of China, Hefei, Anhui 230026, China

[3]Engineering Research Center of the Ministry of Education for Advanced Battery Materials, School of Metallurgy and Environment, Central South University, No.932 South Lushan Road, Changsha, Hunan 410083, PR China

*Corresponding Authors: pinew@ustc.edu.cn; xdren@ustc.edu.cn

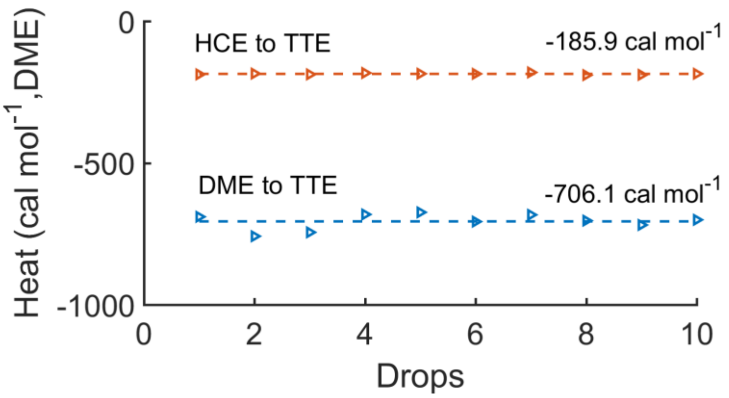

Supplementary Fig. 1. Heat release with HCE to TTE and DME to TTE. To avoid exceeding the instrument measurement range, the volume of DME per droplet was 0.2 μL when added into TTE, and that of HCE was 1 μL. The heat release from HCE to TTE was calculated based on the actual amount of DME in each droplet.

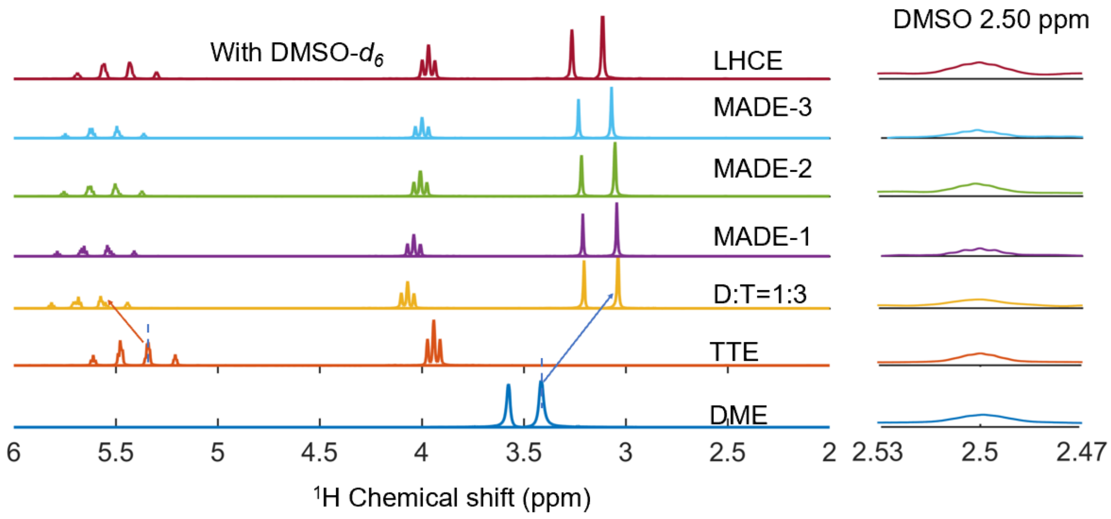

Supplementary Fig. 2. ^{1}H-NMR spectra of different solvents and electrolytes

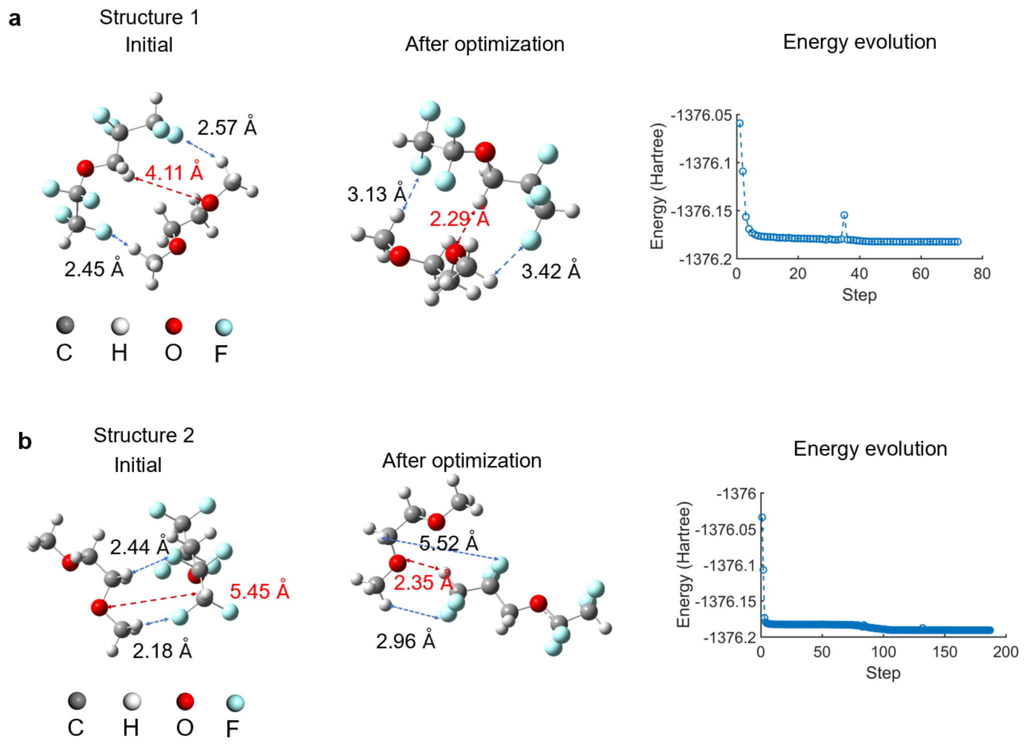

Supplementary Fig. 3. **a-b** DFT optimization of different configurations of the DME-TTE complexes structure 1 (**a**) and structure 2 (**b**) where the $F_{(TTE)}$ is brought into proximity with the $H_{(DME)}$ initially.

We brought $H_{(DME)}$ into proximity with $F_{(TTE)}$ and subsequently performed geometry optimizations using DFT. The optimization results showed that the $H_{(DME)}$ moved away from the $F_{(TTE)}$, while the $H_{(TTE)}$ moved closer to the $O_{(DME)}$. To further verify this result, we selected another structure, and both calculations exhibited similar outcomes. The distances between $H_{(DME)}$ and $F_{(TTE)}$ before optimization were 2.45 Å (structure 1, 2.44 Å in structure 2) and 2.57 Å (structure 1, 2.18 Å in structure2), respectively. After optimization, the distances increased to 3.13 Å (structure 1, 5.52 Å in structure 2) and 3.43 Å (structure 1, 2.96 Å in structure 2). On the other hand, the distance between $O_{(DME)}$ and $H_{(TTE)}$ decreased from the original 4.11 Å (structure 1, 5.45 Å in structure 2) to 2.29 Å (structure 1, 2.35 Å in structure 2). These results suggested that the C-$H_{(TTE)}$…O-C $_{(DME)}$ interactions are favored compared to C-$H_{(DME)}$…F-C$_{(TTE)}$ interactions.

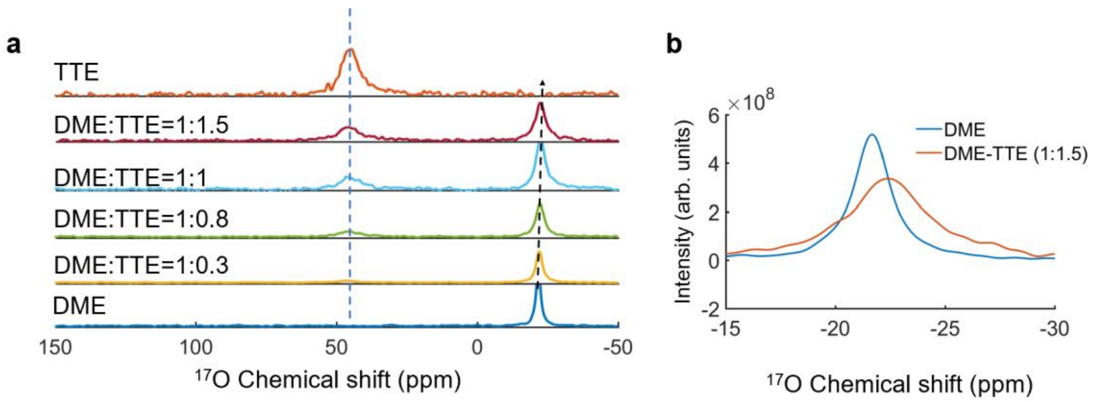

Supplementary Fig. 4. a ^{17}O NMR spectra of mixed solutions with different ratios of DME and TTE. **b** Comparison of ^{17}O NMR spectra between DME and DME-TTE (1:5 in molar ratio)

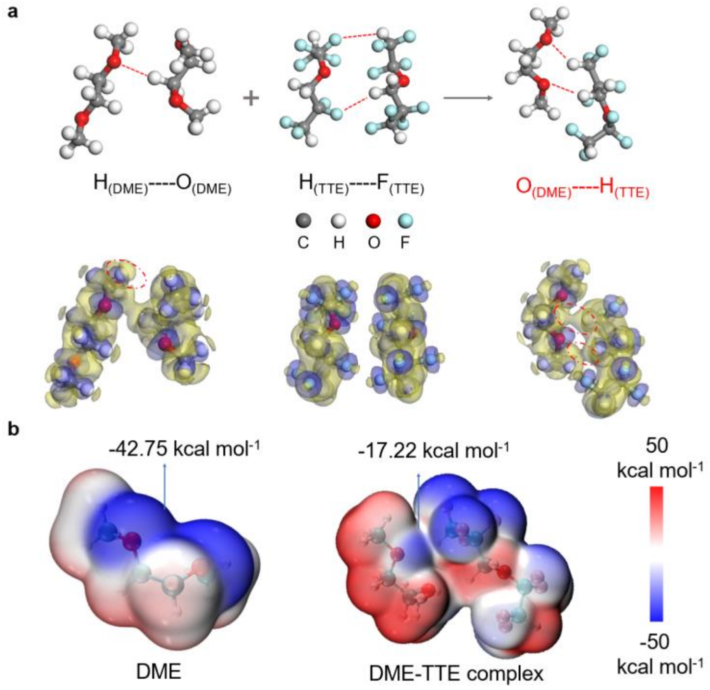

$$Li^{+}(DME)_{X}(FSI^{-})_{y} + xTTE \leftrightharpoons xDME(TTE) + LiFSI\downarrow + (y-1)FSI^{-}$$

Supplementary Fig. 5. a The optimized molecular coordination configuration and plot of deformation electron density of DME-DME, TTE-TTE and DME-TTE complexes. Isosurface: 0.03 a.u, where yellow and purple represent electron loss and accumulation, respectively. **b** The projection of the electrostatic potential onto the electron equipotential surface before and after TTE coordination.

After TTE coordination, the minimum electrostatic potential energy in the vicinity of oxygen of DME increased significantly. Dispersing the polarity of oxygen aids in enhancing the oxidative stability of molecules. This result can also explain the frequently observed experimental phenomenon: the solubility of salts in DME diminishes when combined with TTE, or introducing a diluent to a concentrated electrolyte may induce salt precipitation. This is due to the competition between Li^{+} and diluent for coordination with DME, resulting in the establishment of a coordination equilibrium for DME.

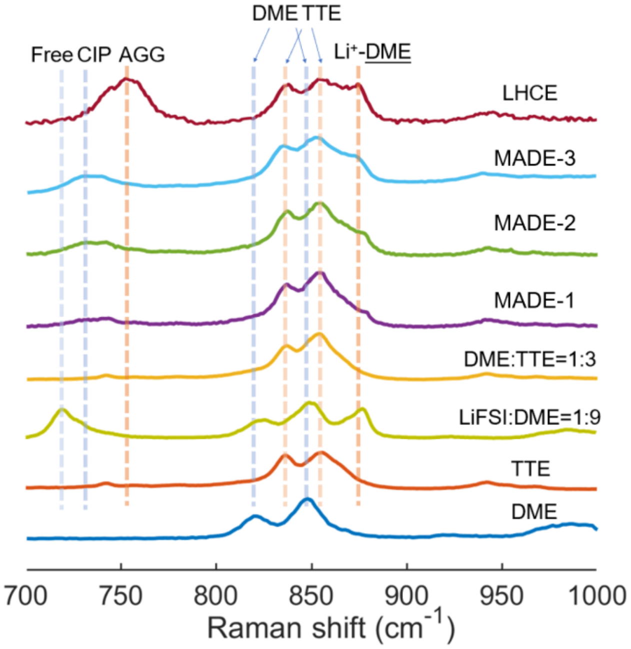

Supplementary Fig. 6. The Raman spectra of DME, TTE, DE, DME-TTE (1:3 in molar ratio), MADE-1, MADE-2, MADE-3 and LHCE.

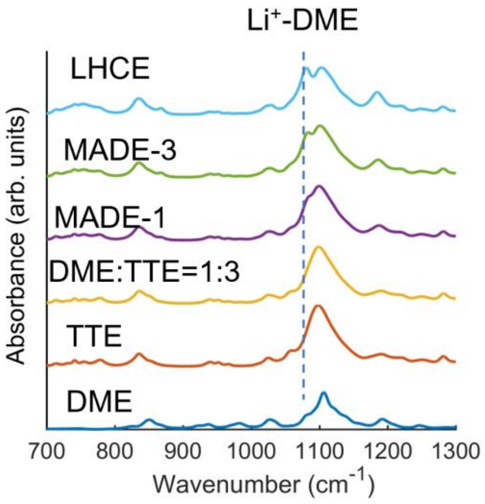

Supplementary Fig. 7. Infrared absorption spectra of DME, TTE, DME-TTE (1:3 in molar ratio), MADE-1, MADE-3 and LHCE.

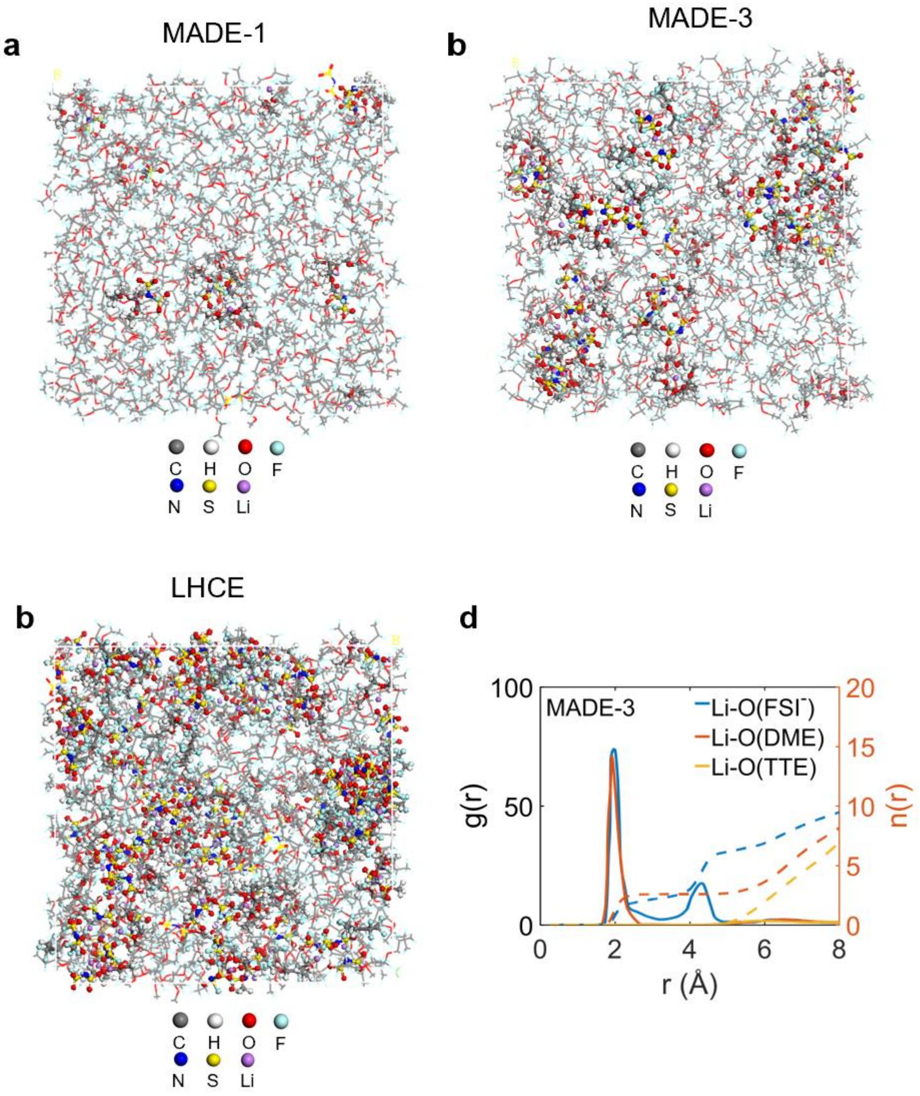

Supplementary Fig. 8. a-c Snapshots of molecular dynamics (MD) simulation of MADE-1 (**a**), MADE-3 (**b**), and LHCE (**c**). (The coordination structure of Li^+ was shown with the ball and stick model and the rest of the molecules were shown with the line model). **d** The radial distribution functions and coordination numbers calculated from MD simulations of MADE-3

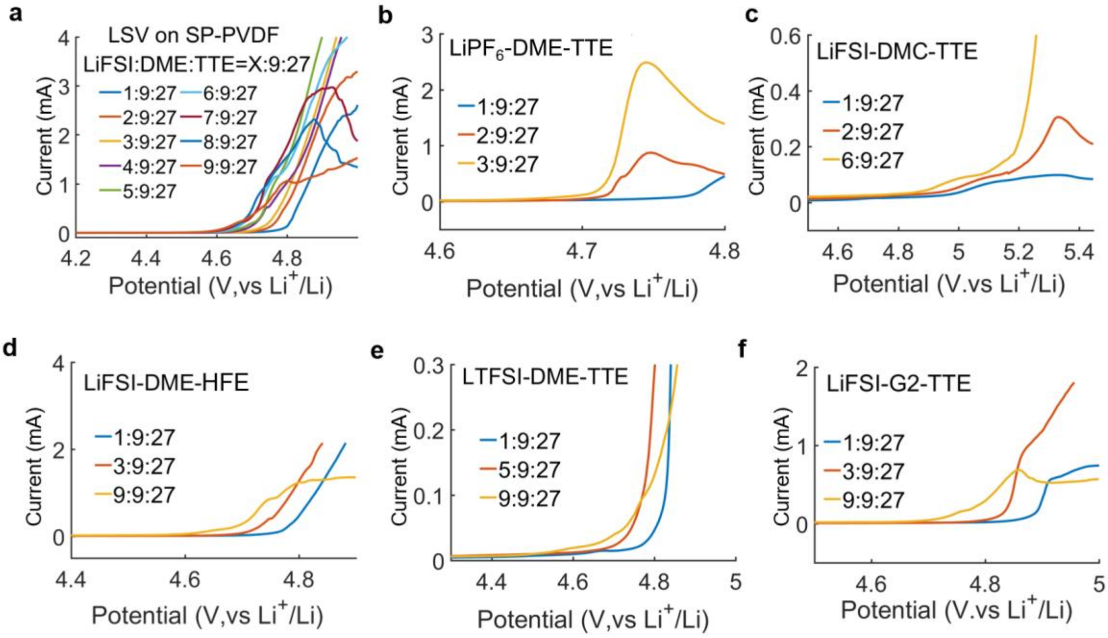

Supplementary Fig. 9. The LSV results for different formulations of electrolytes of LiFSI-DME-TTE (**a**), $LiPF_6$-DME-TTE (**b**), LiFSI-DMC-TTE (**c**), LiFSI-DME-HFE (**d**), LiTFSI-DME-TTE (**e**) and LiFSI-G2-TTE (**f**).

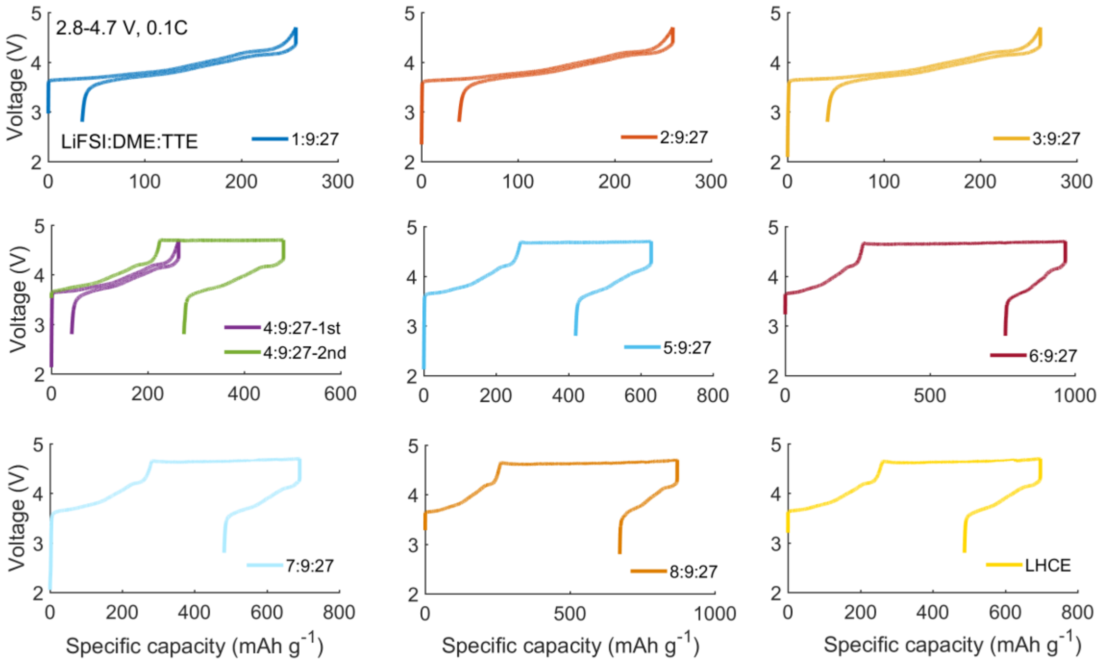

Supplementary Fig. 10. The voltage profiles of Li||NMC cells using electrolytes with different concentrations of LiFSI.

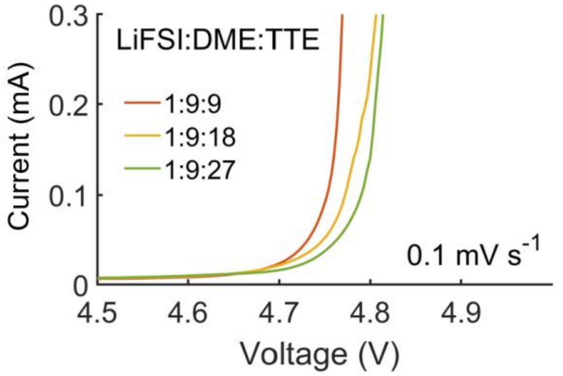

Supplementary Fig. 11. The LSV results for the electrolytes with different TTE ratios using Li||Al (Al foil coated with PVDF-Super P carbon nanoparticles) cells.

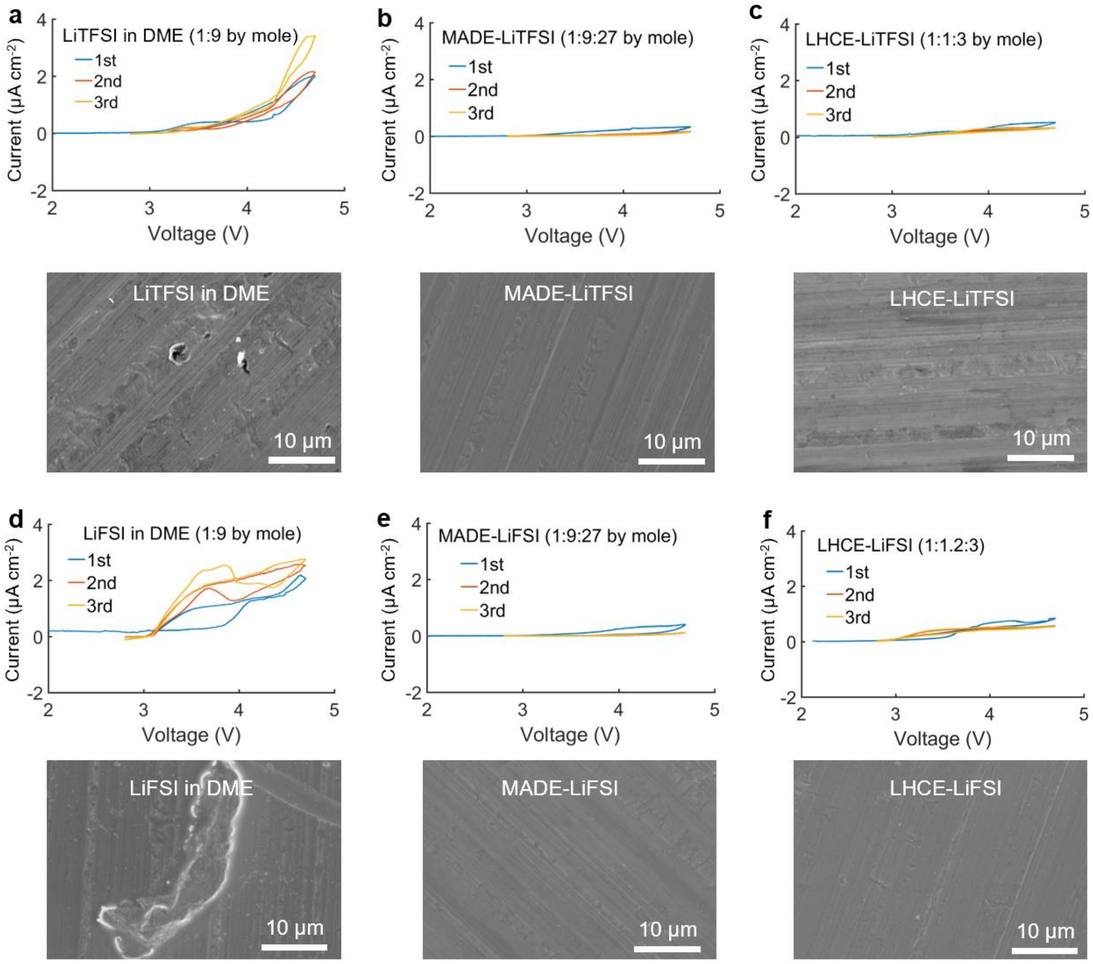

Supplementary Fig. 12. **a-f** The Al corrosion behavior and corresponding SEM morphology after CV scans in different electrolyte formulations of LiTFSI in DME (**a**), MADE-LiTFSI (**b**)LHCE-LiTFSI (c), LiFSI in DME (**d**), MADE-LiFSI (**e**), and LHCE-LiFSI (**f**)

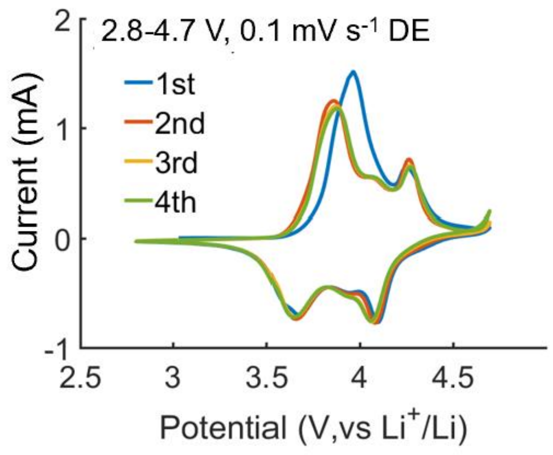

Supplementary Fig. 13. CV curves of Li||NMC cell in DE (LiFSI:DME=1:9 in molar ratio).

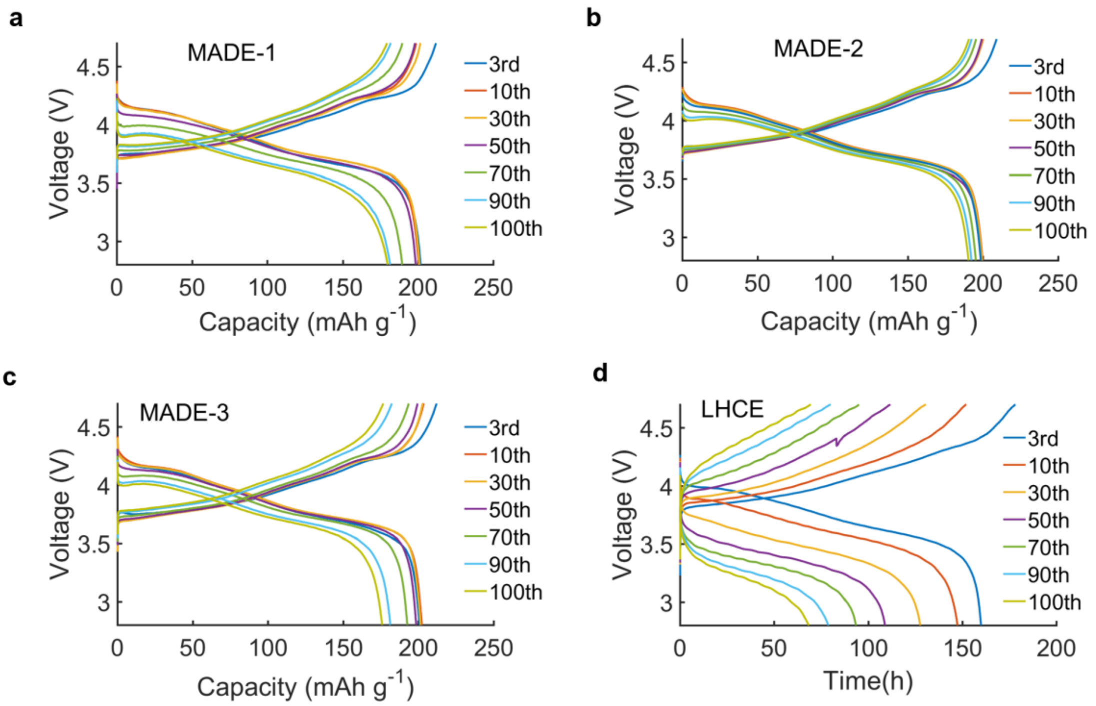

Supplementary Fig. 14. Voltage curves of MADE-1 (**a**), MADE-2 (**b**), MADE-3 (**c**) and LHCE (**d**).

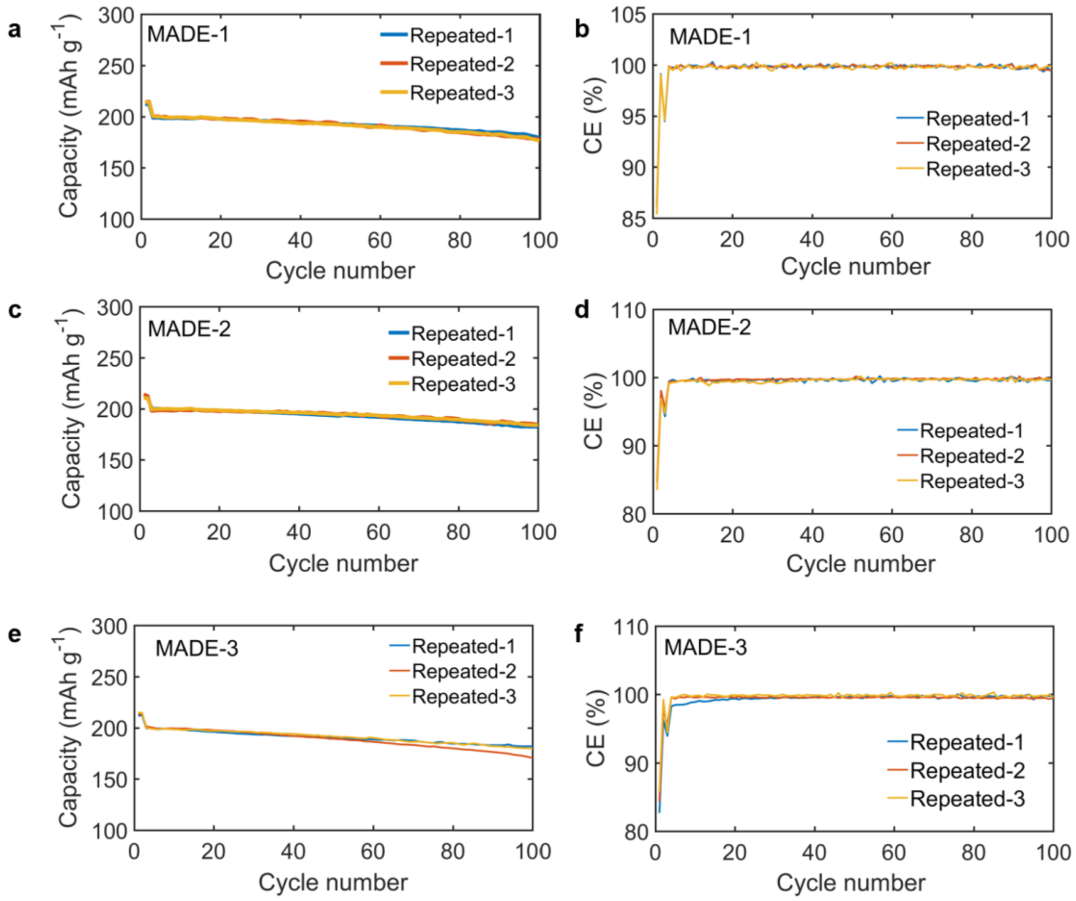

Supplementary Fig. 15. The repeated cycling performance and Coulombic efficiency tests for Li||NMC cells in MADE-1 (**a-b**), MADE-2 (**c-d**) and MADE-3 (**e-f**) between 2.8-4.7 V.

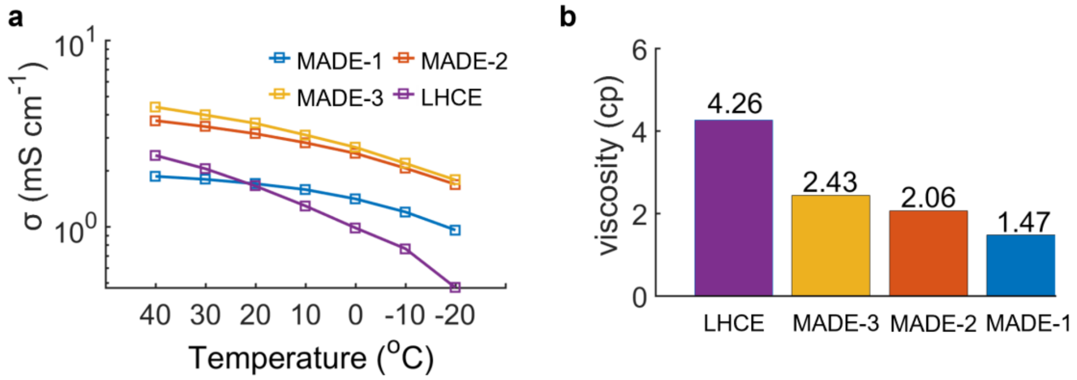

Supplementary Fig. 16. a Temperature-dependent ionic conductivity for MADEs and LHCE. **b** The viscosity of MADE-1, MADE-2, MADE-3 and LHCE.

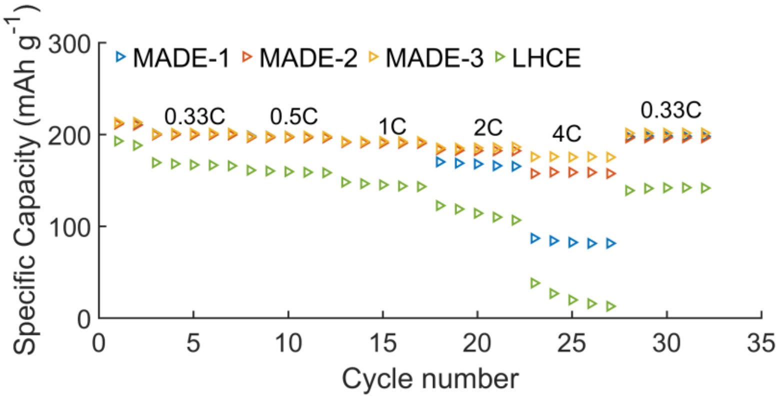

Supplementary Fig. 17. The rate capacity of MADE-1, MADE-2, MADE-3 and LHCE.

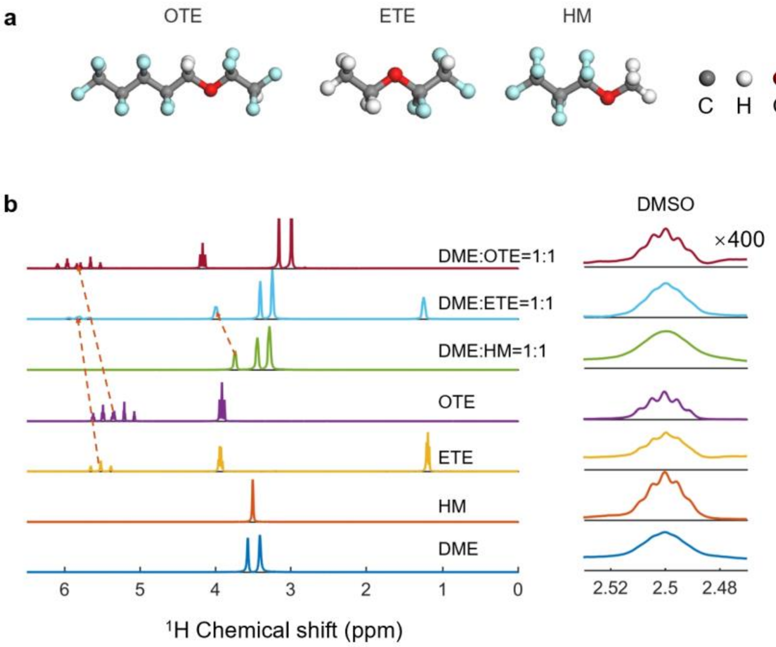

Supplementary Fig. 18. **a** The molecular structures of different hydrofluoroethers (OTE, ETE and HM). **b** The ^{1}H-NMR spectra of different solvents and mixtures.

The ^{1}H chemical shifts of -CF_2H of OTE and ETE are 5.4 and 5.5 ppm, respectively, while that of -CH_3 of HM is 3.5 ppm. After mixing with DME, the -CF_2H signals of OTE and ETE both shift apparently to 5.8 ppm, while the -CH_3 signal of HM barely shifts to 3.7 ppm. This indicates that the anchoring effect between ETE/OTE and DME is stronger than that between HM and DME.

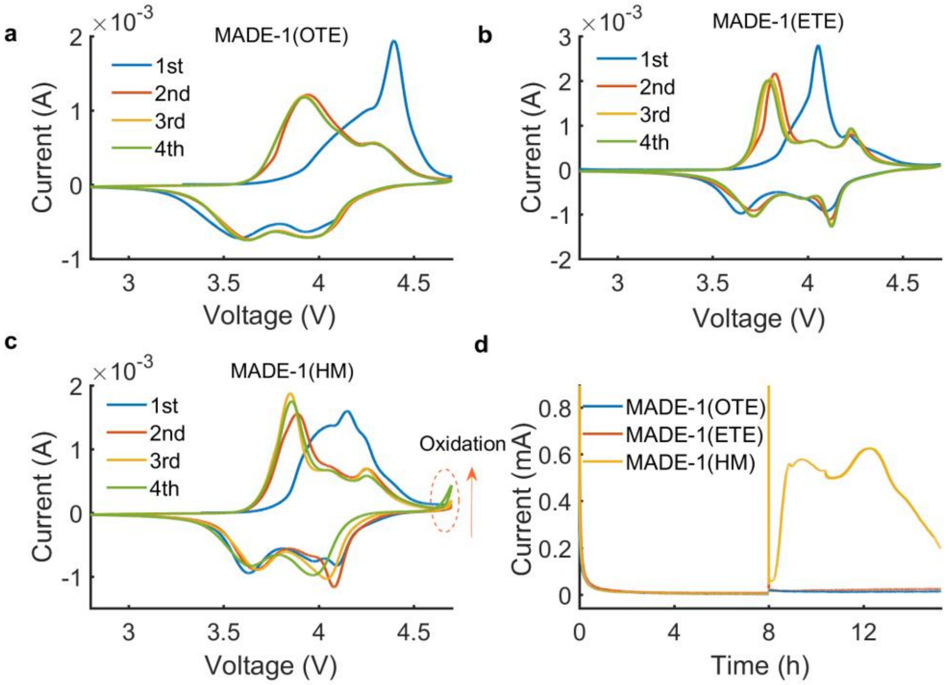

Supplementary Fig. 19. **a-c** CV curves of the cell with different MADEs with OTE (**a**), ETE (**b**) and HM (**c**) as molecular anchors at 0.1 mV s^{-1}. **d** The leakage currents of Li||NMC811 cells with different MADEs at 4.6 V and 4.7 V.

The anodic stability with different MADEs was estimated by CV. The molar ratios of salt, DME, and the anchoring agent are fixed at 1:9:27. MADE-1(OTE) and MADE-1(ETE) exhibit no excessive oxidation side reactions during scans between 2.8-4.7 V, indicating that OTE and ETE with the -CF_2H moiety have similar anchoring effect. In contrast, increased oxidation currents and higher leakage currents were observed with the use of HM, suggesting the unique role of the -CF_2H moiety

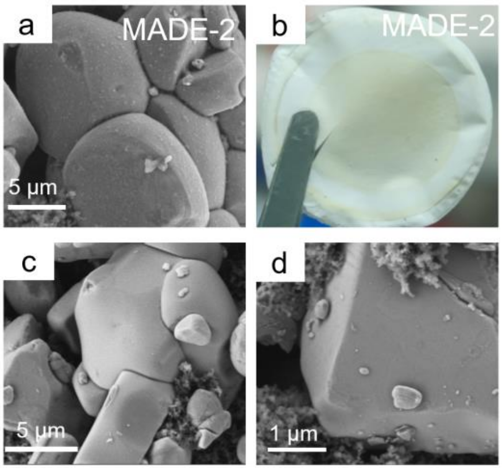

Supplementary Fig. 20. **a** SEM morphology of cathodes recovered from MADE-2 after 50 cycles. **b** Optical image of the separator from MADE-2 after 50 cycles. **c-d** SEM morphology of pristine NMC811.

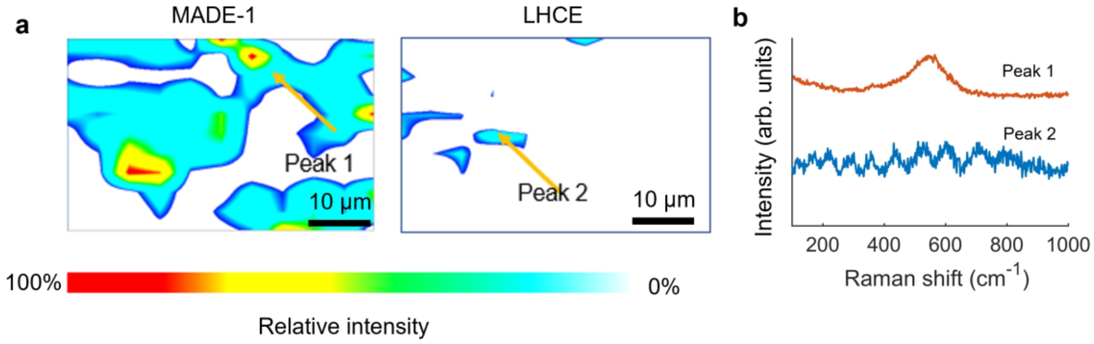

Supplementary Fig. 21. a Comparison of 2D Raman mapping of the cathodes after 50 cycles in MADE and LHCE**. b** Comparison of Raman signals at the position pointed by the arrow in Figure a.

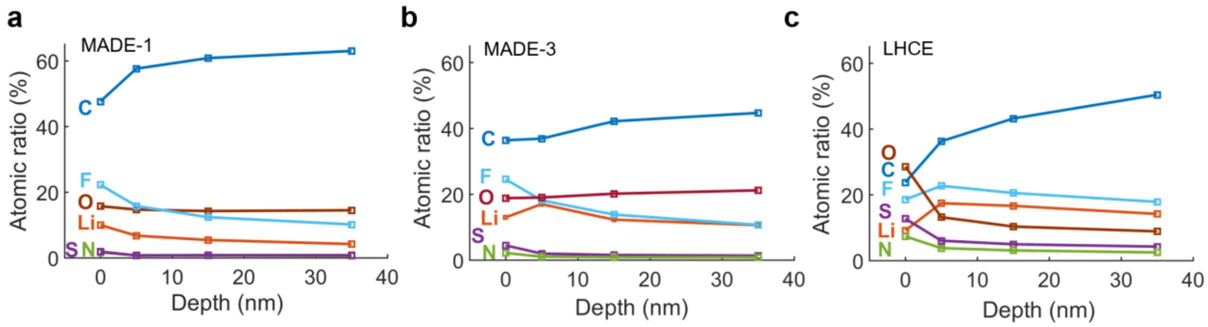

Supplementary Fig. 22. Quantified atomic ratios of CEI for MADE-1 (**a**), MADE-3 (**b**) and LHCE (**c**) from XPS characterization.

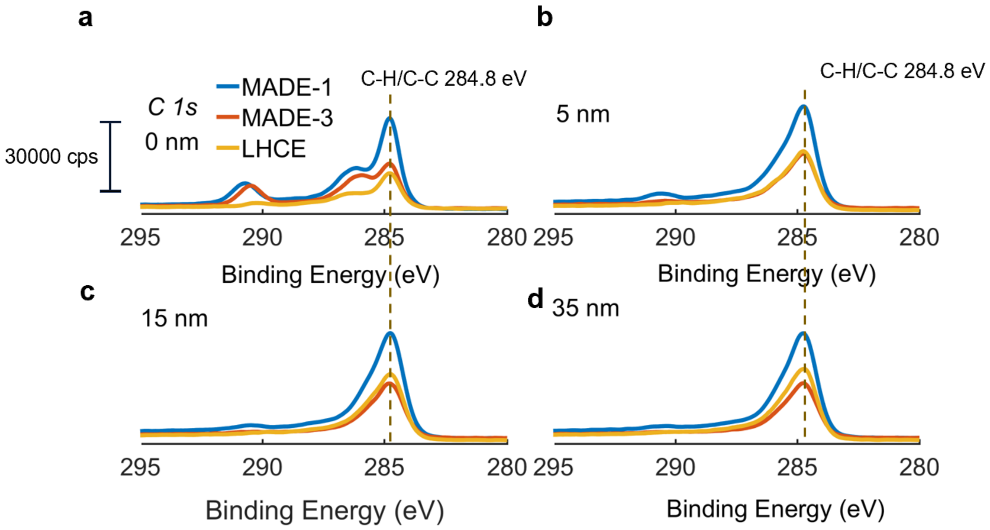

Supplementary Fig. 23. The comparison of XPS *C 1s* spectra after 50 cycles at 2.8-4.7 V for MADE-1, MADE-3 and LHCE at different depths during Ar^{+} sputtering: 0 nm (**a**), 5 nm (**b**), 15 nm (**c**) and 35 nm (**d**).

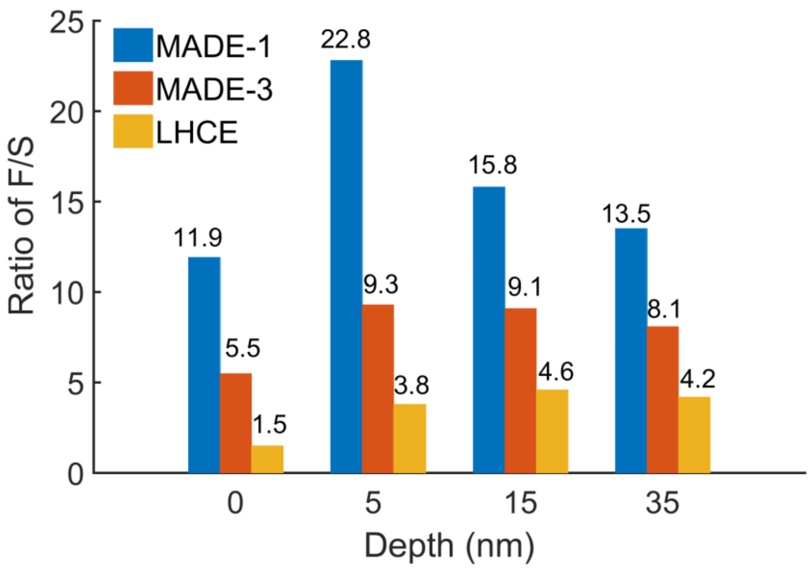

Supplementary Fig. 24. The ratio of F/S for MADE-1, MADE-3 and LHCE from XPS tests at different depths during Ar^+ sputtering.

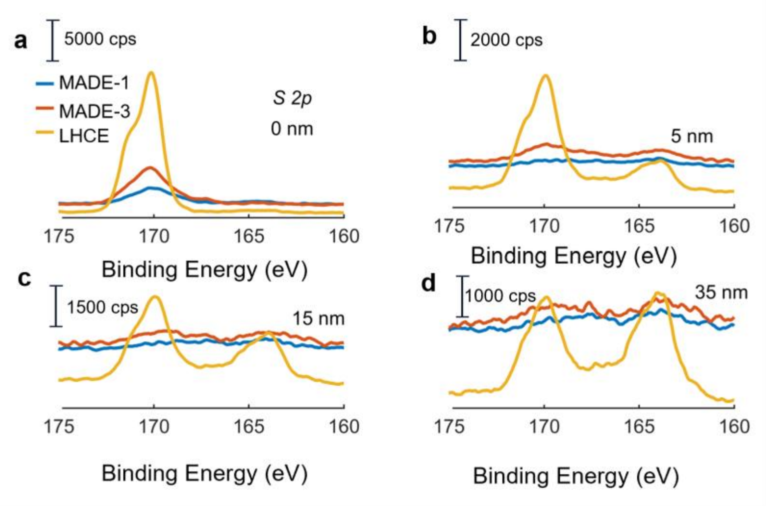

Supplementary Fig. 25. The comparison of XPS *S 2p* spectra on cathodes after 50 cycles at 2.8-4.7 V for MADE-1, MADE-3 and LHCE at different depths during Ar^+ sputtering: 0 nm (**a**), 5 nm (**b**), 15 nm (**c**) and 35 nm (**d**).

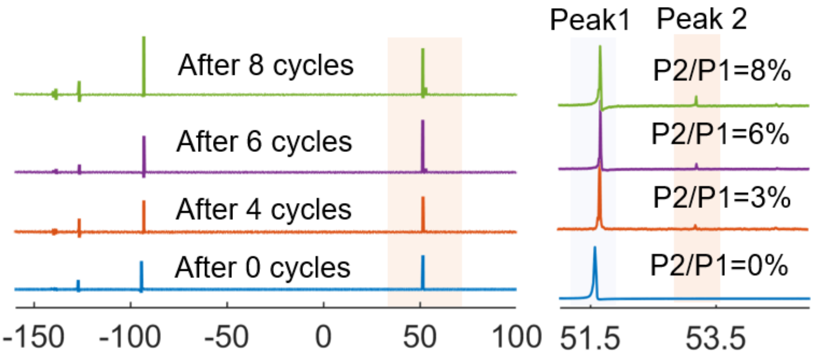

Supplementary Fig. 26. The ex-situ ^{19}F NMR for LHCE after 0, 2, 4, 6, and 8 cycles.

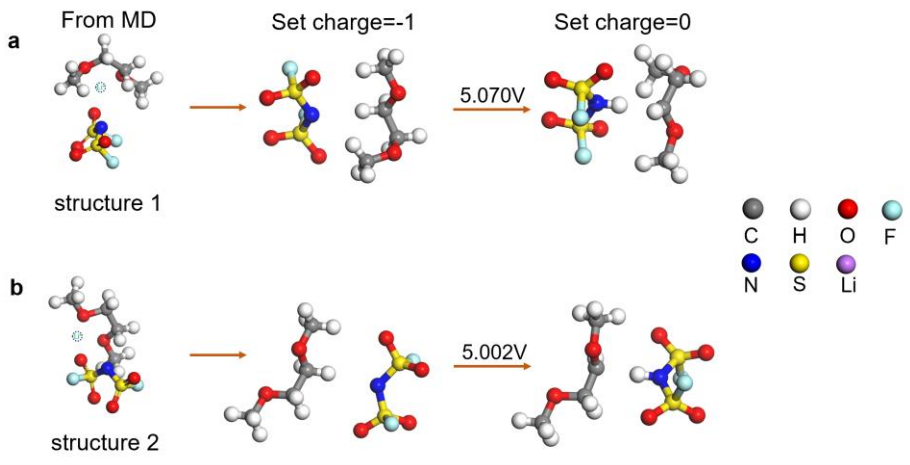

Supplementary Fig. 27. Simulation and optimization of the oxidation process of two different configurations of DME-TTE complexes extracted from MD simulations (structure 1 (**a**), structure 2 (**b**), the circle drawn with dashed lines represents the position of lithium).

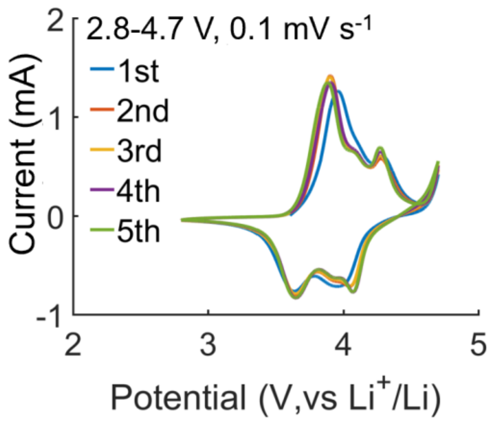

Supplementary Fig. 28. CV curves of the Li||NMC cell with LHCE (the cathode from the cell with MADE-1 after three cycles at 0.1C).

To verify whether the CEI layer formed in MADE-1 could inhibit electrolyte oxidation at 4.7 V. The NMC811 electrode is first cycled in MADE-1, and then the cathode is retrieved for battery assembly using LHCE, followed by CV testing. The result indicated that the CEI cannot prevent the oxidation of LHCE at 4.7 V, implying that the stability of MADE-1 is independent of the CEI.

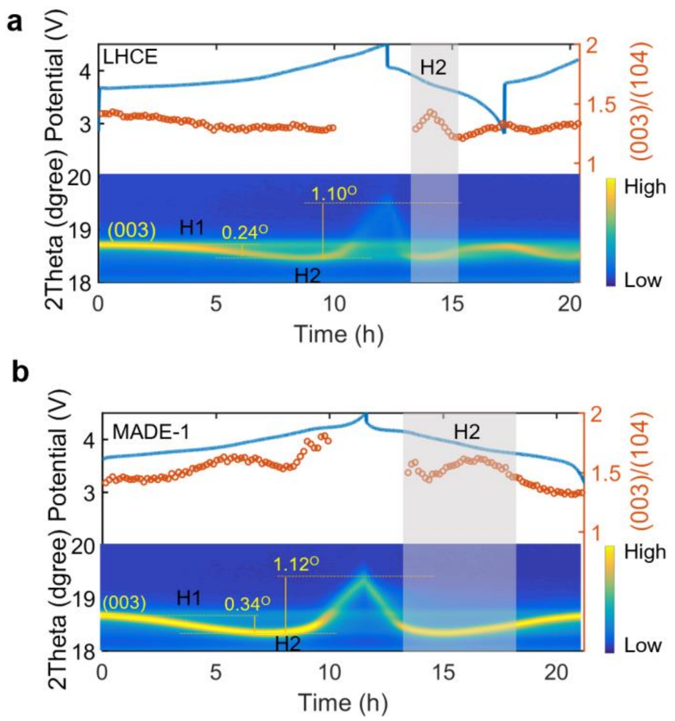

Supplementary Fig. 29. In-situ XRD pattern of the cathode recovered from the cell cycled four times at 0.1C in LHCE (**a**) and MADE-1 (**b**).

In order to investigate the effect of excessive side reactions on the electrode structure, the NMC811 electrode was first activated in MADE-1 and LHCE, respectively, and then the cathode was used for in situ XRD testing. The same carbonate-based electrolyte (1M $LiPF_6$ in EC/DMC/EMC) was used during in-situ XRD testing to characterize the differences caused by the CEI layer. The results indicate that excessive side reactions not only thicken the interface layer of the cathode, but also induce the failure of the electrode structure. This may be due to the irreversible transition of electrode structure towards lithium deficient phase caused by lithium not being embedded in a timely manner or lithium loss caused by side reactions.

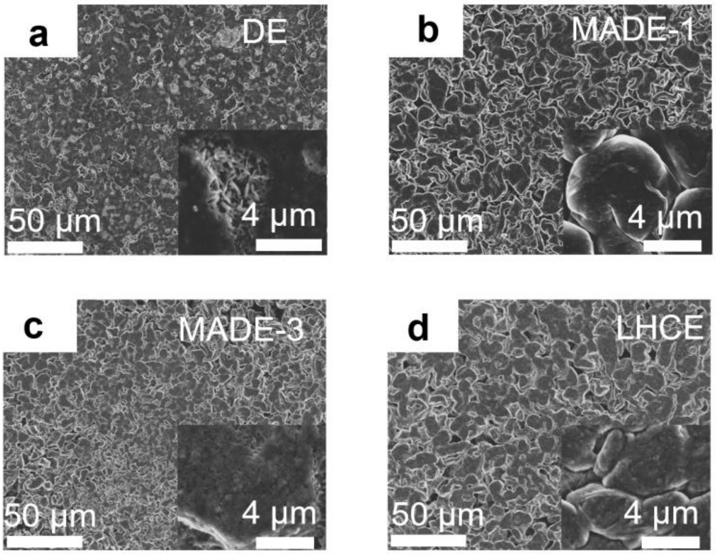

Supplementary Fig. 30. SEM images of Li deposition morphologies of DE (**a**), MADE-1 (**b**), MADE-3 (**c**) and LHCE (**d**) with a current density of 0.5 mA cm^{-2} for 4 mAh cm^{-2} (Insert: Enlarged views of Li deposition morphology).

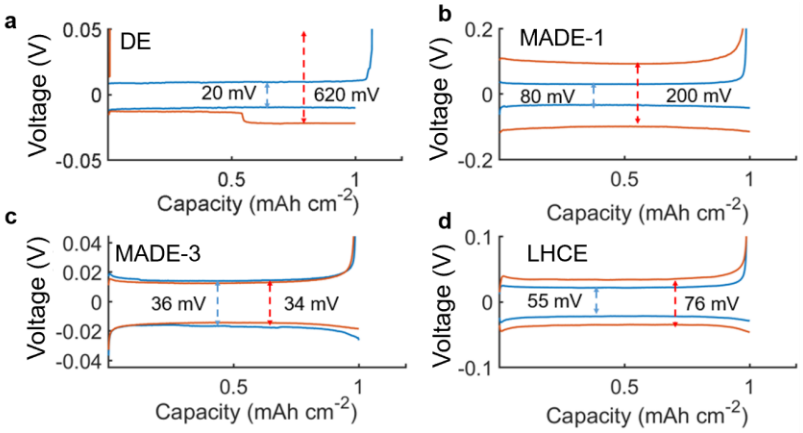

Supplementary Fig. 31. The voltage curves at 20th and 100th cycle of Li||Cu cells with 0.5 mA cm^{-2} and 1 mAh cm^{-2} for DE (**a**), MADE-1 (**b**), MADE-3 (**c**) and LHCE (**d**).

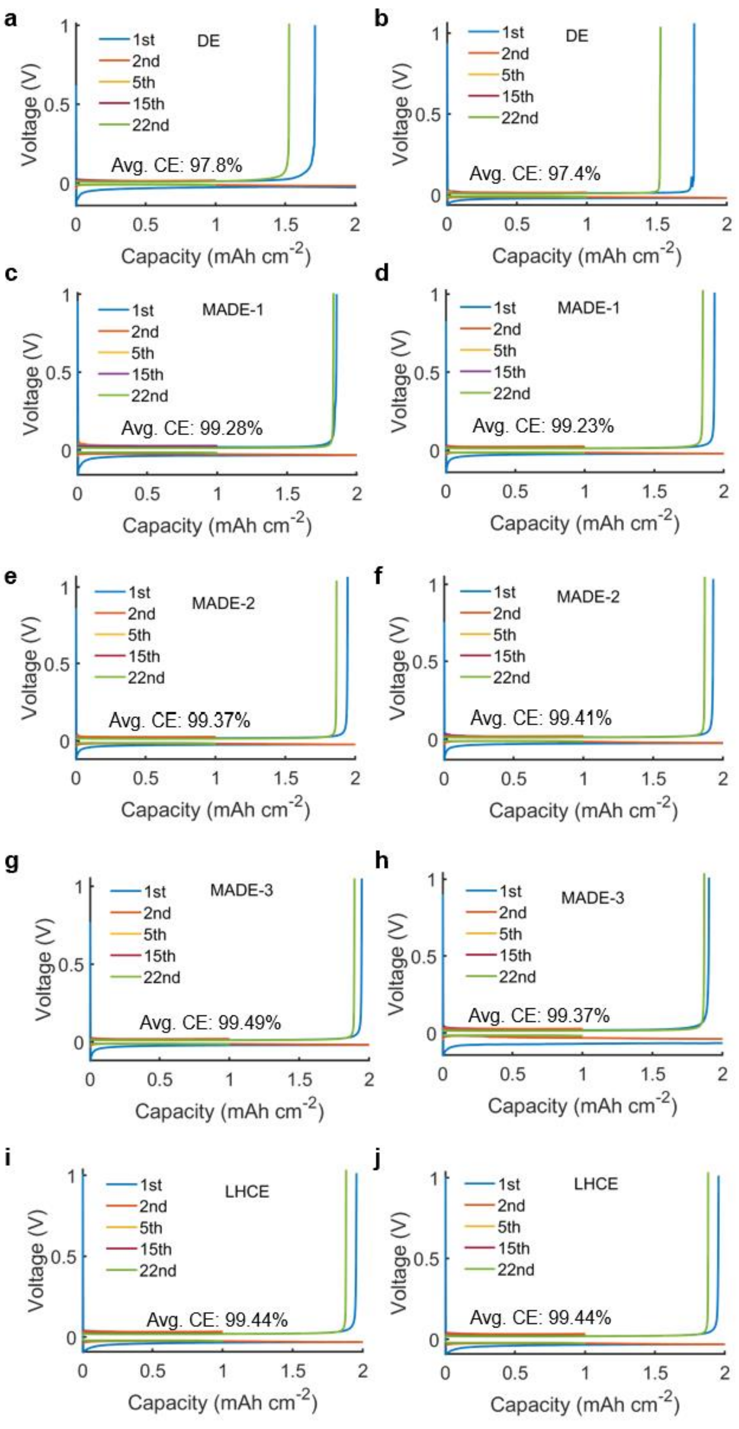

Supplementary Fig. 32. Two parallel data of Li CE tests using Aurbach's method in different electrolytes of DE (**a-b**), MADE-1 (**c-d**), MADE-2 (**e-f**), MADE-3 (**g-h**) and LHCE (**i-j**)

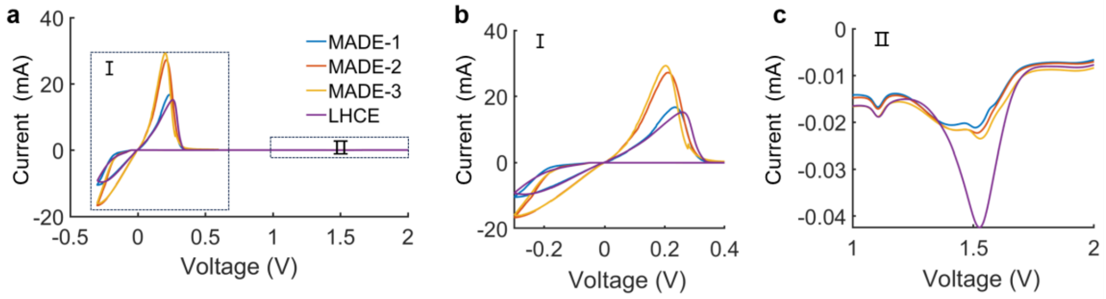

Supplementary Fig. 33. a CV curves of Li plating/stripping in different electrolytes with 5 mV s^{-1}. **b** Enlarged view of region I in Figure a. **c** Enlarged view of region II in Figure a.

The clustering of anions introduced by a high salt content increases the overpotential for Li plating/stripping, and aggravates the consumption of Li metal by reactions with anion. Therefore, excessively high salt concentration is not favorable for improving Li CE.

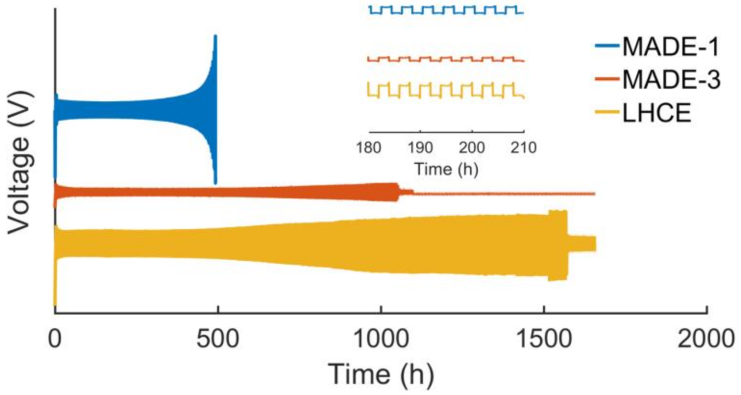

Supplementary Fig. 34. Cycling of Li||Li symmetrical cells using MADE-1, MADE-3, and LHCE (Insert: enlarged view of cycling curves from 180 h to 210 h).

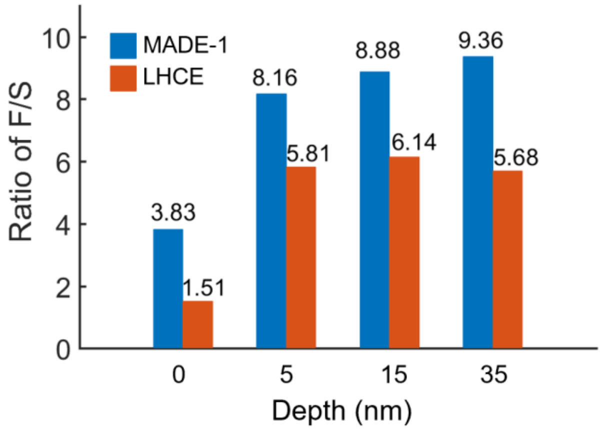

Supplementary Fig. 35. The ratio of F/S of SEI from XPS characterization on Li metal recovered from the Li||NMC811 cells after 50 cycles for MADE-1 and LHCE.

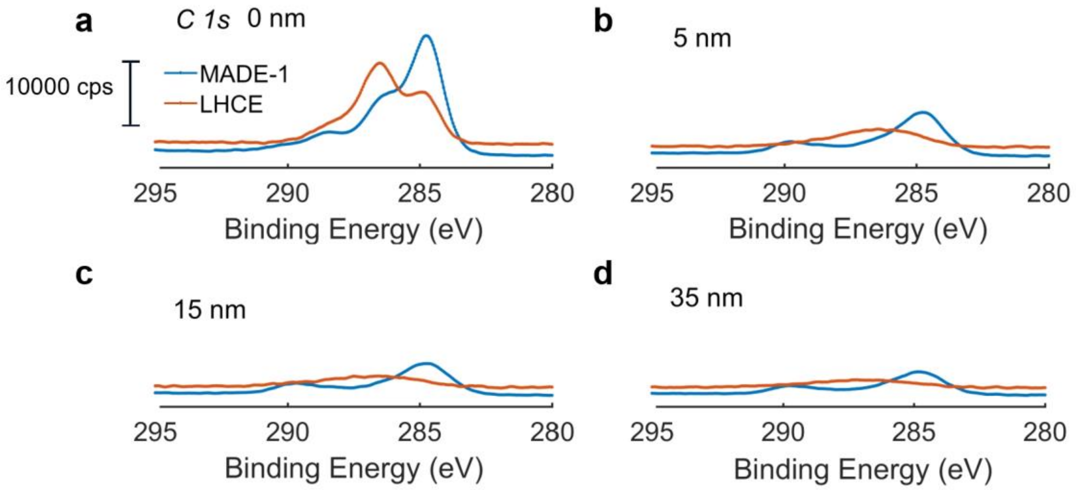

Supplementary Fig. 36. The XPS *C 1s* spectra on anodes for MADE-1 and LHCE at different depths during Ar^+ sputtering: 0 nm (**a**), 5 nm (**b**), 15 nm (**c**) and 35 nm (**d**).

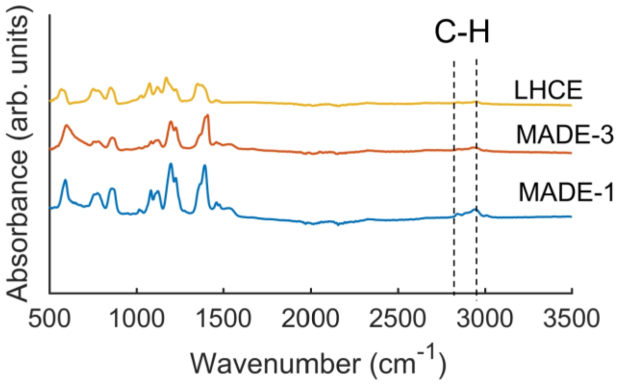

Supplementary Fig. 37. The infrared absorption spectra of the Li SEI layers formed on Cu foils in the LHCE, MADE-3 and MADE-1. All samples were obtained from the Li||Cu cells after 5 plating/stripping cycles.

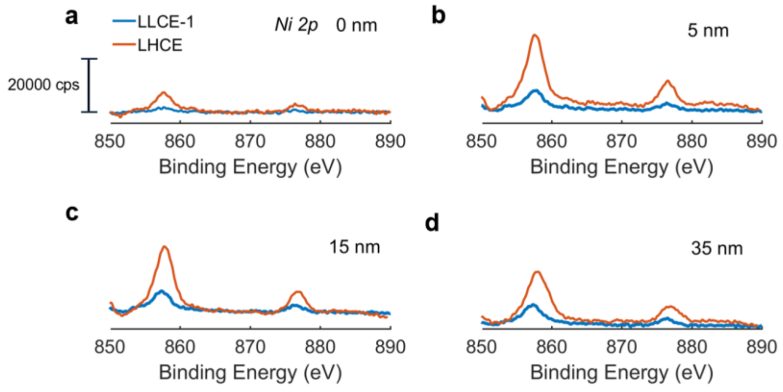

Supplementary Fig. 38. The XPS *Ni 2p* spectra on anodes for MADE-1 and LHCE at different depths during Ar^+ sputtering: 0 nm (**a**), 5 nm (**b**), 15 nm (**c**) and 35 nm (**d**).

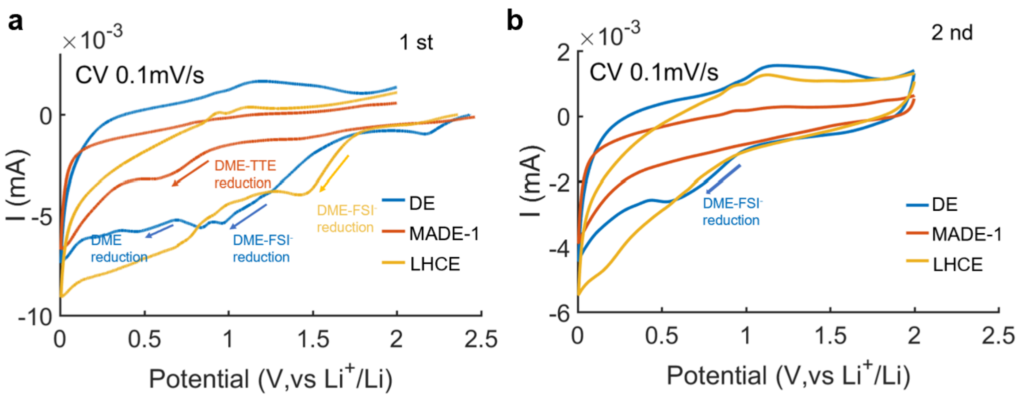

Supplementary Fig. 39. The cyclic voltammetry with a scanning rate of 0.1 mV s^{-1} in a potential range from 2.5 V to 0.0 V of DE, MADE-1 and LHCE for the first cycle (**a**) and second cycle (**b**). The intensity of the LHCE reduction peak and the initial potential are higher than those of MADE-1, indicating that LHCE has stronger reactivity on the anode.

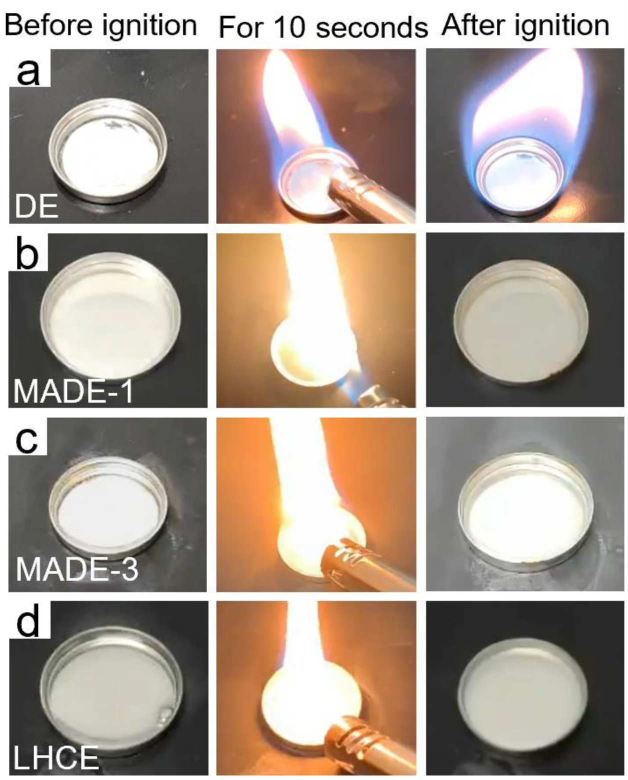

Supplementary Fig. 40. Flammability tests of DE (**a**), MADE-1 (**b**), MADE-3 (**c**) and LHCE (**d**).

Supplementary Table 1. Cell parameters of the Li||NMC811 pouch cell

	Parameter	Value
NMC811 cathode	Capacity	2.1 Ah
	Active material loading	96.5%
	Area capacity (each side)	2.5 mAh cm^{-2}
	Al foil thickness	12 µm
Li anode	total thickness without Cu foil	50 µm
N/P		2.1
Electrolyte	E/C	3 g Ah^{-1}
Separator	PE	12 µm

Reference

1. Lai, P,, Hua, H., Huang, B., Zhang, P., Zhao, J. A Carboxylic Ester-Based Electrolyte with Additive to Improve Performance of Lithium Batteries at Ultra-Low Temperature. *J. Electrochem. Soc.* **169**, 100539 (2022).

2. Han, J.G., *et al.* Unsymmetrical fluorinated malonatoborate as an amphoteric additive for high-energy-density lithium- ion batteries. *Energ. Environ. Sci.* **11**, 1552-1562 (2018).

3. Gibson, L.D., Pfaendtner, J. Solvent oligomerization pathways facilitated by electrolyte additives during solid-electrolyte interphase formation. *Phys. Chem. Chem. Phys.* **22**, 21494-21503 (2020).

4. Ren, X. *et al.* Role of inner solvation sheath within salt – solvent complexes in tailoring electrode/electrolyte interphases for lithium metal batteries. *Proc. Natl. Acad. Sci. U.S.A.* **117**, 28603-28613 (2020).

5. Lu, T., Chen, F. Multiwfn: A multifunctional wavefunction analyzer. *J. Comput. Chem.* **33**, 580-592 (2012).

6. Chowdhury, P.K. Infrared depletion spectroscopy suggests fast vibrational relaxation in the hydrogen-bonded aniline-tetrahydrofuran (C_6H_5-NH_2 ··· OC_4H_8) complex. *Chem. Phys. Lett.* **319**, 501-506 (2000).
