# Supplementary material for: Molecular anchoring of free solvents for high-voltage and high-safety lithium metal batteries
Source: Nat Commun. 2024 Mar 6;15:2033. doi: 10.1038/s41467-024-46186-y (PMC10918083; doi:10.1038/s41467-024-46186-y)
Supplement: Supplementary file 3 — Description of Additional Supplementary Files [file 41467_2024_46186_MOESM3_ESM.pdf]

DESCRIPTION OF ADDITIONAL SUPPLEMENTARY FILES DOCUMENT

Supplementary Movie 1. Flammability test of DE (LiFSI9DME).

Supplementary Movie 2. Flammability test of LHCE (LiFSI-1.2DME-3TTE).

Supplementary Movie 3. Flammability test of MADE-1 (LiFSI-9DME-27TTE).

Supplementary Movie 4. Flammability test of MADE-3 (3LiFSI-9DME-27TTE).
